# Image of the Family and Orienting Image of Attachment: A Psychosemantic Approach

**DOI:** 10.11621/pir.2025.0411

**Published:** 2025-12-15

**Authors:** Apollinaria V. Chursina

**Affiliations:** a Lomonosov Moscow State University, Russia; b University of Granada, Spain; c Federal Scienti! c Center for Psychological and Interdisciplinary Research, Moscow, Russia

**Keywords:** romantic attachment, image of family, orienting image of attachment, mate preferences

## Abstract

**Background.:**

There are genetic, social, and interpersonal factors that determine the relationship between mate preferences and parental figures. Attachment theory offers an explanatory principle about the continuity of relationships with the primary caregiver and romantic partner.

**Objective.:**

To integrate the cultural-historical and attachment approaches, and to examine the contribution of attachment style to the image of the family, as well as to conceptualize the orienting image of attachment.

**Design.:**

237 heterosexual Russian individuals participated in the study. The Experiences in Close Relationships-Revised (ECR-R) Questionnaire and a Semantic Dif erential Scale were used to assess attachment style, as well as images of self, romantic partner, mother and father, and the family.

**Results.:**

Signiicant dif erences in the image of the family were found depending on attachment style; however, no differences were found in the image of the parental family. Attachment insecurity dimensions are associated with perceptions of the similarity of image of the mother and the romantic partner image in men and women (both dimensions for women and avoidance for men). Characteristics of the mother and father images predict attachment anxiety in women, while the father image predicts attachment avoidance in men.

**Conclusion.:**

The orienting image of attachment was conceptualized as the image of the mother, which is explained by the cultural patterns of upbringing in the Russian sample; however, individual characteristics of parental images act as predictors of attachment insecurity dimensions.

## Introduction

### Romantic Attachment Conceptualization: Dynamic Homeostasis of the Images of Self and Significant Other

Attachment theory goes back to the works of J. Bowlby and M. Ainsworth (*e.g.*, Bowlby, 1982), which postulated that the fi rst experience of interpersonal relationships with the primary caregiver makes a contribution to personal development later reflected in relationships with the object of adult romantic attachment (Bartholomew, & Horowitz, 1991). C. Hazan and P. Shaver (1987) argued that romantic love is a manifestation of the attachment system. Th ey observed some similarities between parent-child attachments and romantic relationships, and highlighted that the behavioral system of attachment does not disappear in adulthood—on the contrary, it is used to build the bonds in adult relationships. Several studies conirmed the three types of attachment in adults (e.g., Collins & Read, 1990; Feeney & Noller, 1990), identical to those discovered by the group of Bowlby and Ainsworth.

Subsequently, another typology of romantic attachment was developed (Bartholomew & Horowitz, 1991). It was determined by combinations of two orthogonal dimensions: the image of the self and the image of the significant other, later defined as anxiety and avoidance (Fraley et al., 2000). These were described as anxiety caused by a threat to the attachment system (dimension of anxiety or self-image), and on the other hand, the need to maintain a certain distance or independence in relation to the igure of attachment (dimension of avoidance or model of the other). More precisely, the attachment anxiety measures reflect an individual’s predisposition to a state of alertness in the face of possible signs of rejection or separation from the object of attachment, while the avoidance dimension reflects discomfort or rejection in the face of intimacy or emotional dependencies, or indifference to situations that require closeness with others.

These manifestations are associated with the degree to which a person perceives him- or herself and others as deserving of the love, support, and trust of other people. In this way, four types of attachment are defined: (1) secure, a positive image of oneself and others, characterized by confidence in one’s value to be the object of love and ideas about others as accepting and loving, (2) fearful-avoidant (hereinafter “avoidant”), a negative image of oneself and a negative image of others; isolated in mind with a fear of being rejected, which aggravates the rejection of oneself; (3) preoccupied (hereinater “ambivalent”), a negative image of oneself and a positive image of others; achieves self-acceptance due to a positive assessment of oneself by others; (4) dismissing-avoidant (hereinafter “dismissing”), a positive image of oneself and a negative image of others; independent and avoiding disappointment by refusing close relationships.

### Romantic Attachment and Relationship Functioning: Orienting Image of Attachment and Mate Preferences

Many scholars argue there is an assumption about the continuity of childhood attachment in relation to romantic attachment in adulthood; however, strictly speaking, the few longitudinal studies that exist do not allow us to speak of a strict prediction (e.g., Beijersbergen et al., 2012; Weinfield et al., 2004). The set of images developed in the process of reinforcing repetitive patterns of relationships with the primary caregiver, determines internal working models of attachment which shape the relationship with the object of attachment, and are understood as the general expectations we have about personal relationships. These models consequently act as a kind of prism; they represent a system of attitudes and predispositions regarding oneself and new relationship experience. Hence, different attachment styles represent different types of prisms, or combination of images, that mediate the perception of and interactions with others.

The problem of mate preferences and its connection with parental figures is mul-tifactorial, and studies have shown the importance of genetic, social, interpersonal, and other factors (*e.g.*, Lin et al., 2022; Schulz et al., 2023; Walter et al., 2020; Zhang & Chen, 2020; Zhao et al., 2023). The influence of the parental image on the choice of a romantic partner concerns physical characteristics and characteristics of interaction, and also emotional patterns, but the question of the influence of the image of the parent of one’s own and the opposite sex and gender differences in this matter remains open. For example, a study by Heffernan et al. (2019) confirms both ethological and psychoanalytic assumptions and shows that the inluence of parents is rather generalized, since the connection with the choice of a partner similar to the opposite-sex parent is weak. At the same time, people who have a positive experience of relationships with their parents are more likely to choose partners similar to them. Kocsor et al. (2016) showed that emotional closeness with parents may be important in shaping these preferences; the influence of parental similarity on partner choice may be more pronounced for the opposite-sex parents and depends on emotional relationships in childhood. It should also be emphasized that even in case of phenotypical mate preferences, there is no clear genetic or social explanation for such imprinting, since, for example, women who grew up in a positive family environment more often chose partners similar to their adoptive fathers (Bereczkei et al., 2004).

In this work we will focus on the attachment approach, so we emphasize the concept of the orienting image of attachment as an image of a parent (that is, a system of ideas and attitudes regarding interaction with him/her, as well as about his/her role in the family system), as most significant for the development of the image of a significant other. However, despite a number of studies on the importance of similarity or difference between the image of the significant other and the self-image for mate preferences (e.g., Klohnen & Luo, 2003), it seems crucial to identify the connection of parental images with the image of the significant other and to define the concept of orienting image (the image of a parent that influences on the choice of a romantic partner). However, it remains an open question whether the orienting image is the image of the mother, who is most often considered as the primary caregiver (notably in Russian culture), and so we return to the nature of the attachment concept, which postulates that the emotional connection with the mother and the romantic partner is similar in content, as it satisfies the need for intimacy (albeit slightly different in its nature) and uses the attachment object as a secure base. Nevertheless, the image of the opposite-sex parent can also be considered as orienting, since, following the psychoanalytic tradition, it can be described as a secondary choice of the sexual object which is a romantic partner as signiicant other.

### The Image of the Family

The interaction with parents and correspondent emotional patterns are reflected as the child’s personal meanings and ideas about personal relationships. The family context also reflects culturally specific norms and values. These experiences are imprinted and shape schemes of behavioral patterns, where the image of the world (Leontiev, 1979) is both the initial and the end point of this reflection, since it is a prism which expresses a set of life experiences. Within the framework of the cultural-historical approach (L.S. Vygotsky, A.N. Leontiev, P.Ya. Galperin, etc.), it is possible to consider the image of the family developing the category “the image of the world”. Its nature is social, both in the sense of origin through the development of the psyche in activity, and in the sense of relecting reality and highlighting its essential characteristics (Smirnov, 1985). The orienting function of the image (Galperin, 1976) is primary in relation to activity and acts as an orientation in present situations. Th e image is immanently present in a person’s mind and represents guidelines in the course of a particular activity. The image of the world is a system of meanings, which is integral and develops on the basis of signs that are essential for the person’s activity and experience. The image of the world is not a subjective evaluation; on the contrary, it represents a reflection of reality with meanings, which will be a prism for perception in the future. Therefore, not only cultural, historical, and social contexts are emphasized in this image, but also the individual’s life trajectory. The image of the world is both a phenomenon and a process; it is a dynamic system with its own structure, and has both regulation and orientation functions.

In this work, we will rely on the fact that the image of the family is understood as a system of unconscious attitudes, ideas, and expectations regarding family life in general; this image has an individualized personal character by analogy with the image of the world. We believe that such a separation of concepts will help to avoid confusion in the definition of constructs that are close in content, and will also allow us to clarify the systems of meaning being studied. As a set of unconscious attitudes, ideas and expectations regarding the family, the image of the family represents a prism that, by analogy with the internal working models of attachment, is developed in the course of life experience and further reciprocally determines it; that is, one way or another, it gives a certain predisposition regarding scenarios for the close relationships in view of its speciic content.

The development of balance in a couple requires not only an attunement of individual characteristics and mental representations, but also balancing between two family histories, distribution of functions, and role hierarchy. Of particular interest are actual interactions as stable patterns of behavior repeated through generations, so the development of a sense of “we” in a couple relects the family history and affective stereotypes of interaction of both spouses. In the psychoanalytic sense, the identification of a child with the parent of the same sex and the introjection of superego leads to the development of attitudes towards the processes in dyadic relations (Freud, 1922), and also establishes typical behavioral patterns. Within the framework of attachment theory (Bowlby, 1982), the development of internal working models represents a result of imprinting behavioral patterns of a close adult. An intergenerational tendency of stable behavior patterns within family relationships is also expressed, which is consistent with social learning theory (Bandura & Walters, 1977).

Therefore, the assimilation of the experience of relationships in the family in early childhood is somehow connected with the development of the image of the family— that is, the system of unconscious attitudes, ideas, and expectations regarding family life. Nevertheless, our question is focused on the role of the image of a particular parent, of the same or the opposite sex, and in particular, his or her role in the development of dyadic dynamics and the family unconscious, and expressively, the romantic attachment style. Th e characteristics of the concept of the image, developed within the framework of cultural-historical psychology, emphasizes its social nature, which gives us the possibility to conjoin it with attachment theory, where the system of ideas and attitudes about interpersonal relationships developed within the assimilation of experience is called internal working models.

### The Present Study

The main goal of this study is to draw a parallel and build a connection between the cultural-historical approach and attachment theory, as two approaches that postulate the importance of social experience for the development of personality, as well as mental representations that serve as a prism for perception and building interpersonal relationships in adulthood.

In order to develop this approach in line with family psychology, we see as separate tasks (1) to compare the image of the family in individuals with different romantic attachment styles (by analogy with the components of the internal working model of attachment, the images of self and significant other, which are different for each type of attachment) and stress the primacy of attachment style in relation to the image of the family; and (2) to emphasize the concept of the orienting image of attachment for men and women and thus build these ideas into the problem of continuity of attachment.

In the light of these tasks, we have identified a number of hypotheses. (1) Th e image of the family, by analogy with the image of the self and the image of the significant other, will be different for different types of attachment, since it is a dynamic reflection of the perception of affective interaction in the family. (2) The image of the family, as the most relevant reflection of the romantic relationship that is unfolding at a given moment in a person’s life, will be similar to the image of the parental family, but will be different, depending on the romantic attachment styles. (3) The image of an ideal family (which we describe as “my family in 10 years”) will also be different for the individuals with different romantic attachment styles and will mean a certain increase in positive characteristics. (4) The orienting image of attachment will be the image of the parent of the opposite sex as corresponding in a psychoanalytic sense to the image of a signiicant other, that is, it will be its “prototype.”

In this study, we use a psychosemantic approach, since this instrument is more projective and makes it possible to identify systems of unconscious representations regarding personal relationships, while indirectly causing less psychological resistance and personal conlict than traditional self-report measures.

## Methods

### Participants

The study involved 237 heterosexual individuals aged 18-54 years (M = 30.12, *SD* = 7.40), 76 men (32.06%) and 161 women (67.93%). All respondents were married or lived with a partner. 74.3% had higher education.

### Instruments

The *Experiences in Close Relationships-Revised (ECR-R) Questionnaire* (Fraley et al, 2000; adapted for Russian by Chursina, 2022) assesses romantic attachment using a two-dimensional model of individual differences of romantic attachment. It contains 36 items related to “anxiety” or “avoidance” scales. The participants evaluated statements related to romantic relationships using a 7-point scale ranging from 1 (completely disagree) to 7 (completely agree).

The *Semantic Differential Scale* consisted of 10 pairs of adjectives to evaluate oneself, one’s romantic partner/spouse, parents, and family using a scale ranging from -3 to 3: light - dark, flowing - impetuous, warm - cold, tight - plastic, quiet - loud, reliable - unpredictable, soft - solid, steady - volatile, thick - liquid, calm - unquiet.

### Procedure

All study participants were informed about its objectives and gave voluntary informed consent to participate. Data was collected online.

## Results

### Image of the Family in Individuals with Different Romantic Attachment Styles

To identify the differences in the image of the family in adults with different romantic attachment styles, we used one-way analysis of variance ANOVA (multiple comparisons, Tukey’s HSD test).

#### Comparison of Securely-Attached Individuals with Avoidant Ones

Individuals with secure attachment perceive their families as signiicantly lighter than those with avoidant attachment (HSD = -.81, *p* < .01). Th e avoidance factor in the two-dimensional model is consistent with a negative image of others, which is reinforced by the negative model of the self (anxiety dimension); therefore, the family is seen darker. Individuals with secure attachment see their families as signiicantly more reliable (HSD = -.85, *p <* .01) and significantly more stable (HSD = -.92, *p* < .01) than those with avoidant attachment. Avoidant attachment, which is expressed in a negative attitude towards both oneself and others, has a general adverse attitude in interpersonal relationships. This type of attachment transparently reflects that dynamic feedback where negative experiences due to impaired communication reinforce the negative dimensions of the internal working model, since both dimensions of attachment insecurity are present here. A significant difference on the “thick — liquid” scale (HSD = -.87, *p <* .01) expresses the fluidity of roles and the diffuseness of the boundaries of the family system. Anxiety in interpersonal relationships enhances the perception of such phenomena, if any, and gives a sense of the great significance of any changes that occur in the family.

#### Comparison of Securely Attached Individuals with Ambivalent Ones

Individuals with secure attachment see their families as signiicantly more reliable (HSD = -1.41, p < .00) and significantly more stable (HSD = -1.22, p < .00) than those with ambivalent attachment. The family’s great unpredictability for ambivalent attachment is due to anxiety in interpersonal relationships, which are often like a “battlefield” for them. Th e perceived great volatility of the family for ambivalently attached individuals reflects both inconsistency in relationships and, in general, high sensitivity to changes; their attitude in interpersonal relationships can be compared to a “radar”. Individuals with ambivalent attachment see their family as more luid, compared to securely attached individuals (HSD = -1.01, p < .02). This scale describes to a greater extent the rigidity (and in this case, lexibility) of the hierarchy of the family system, as well as its boundaries. Here, in our opinion, there is an expression of both the changeability of the ambivalent personality and the tendency to get closer to signiicant people. Ambivalently attached individuals perceive the family as significantly more restless than securely attached ones (HSD = -1.17, p < .03). This relects both their increased sensitivity and inconsistency, the perception of relations as constantly unstable.

#### Comparison of Securely Attached Individuals with Dismissing Ones

Individuals with secure attachment see their family as significantly lighter than those with dismissing attachment (HSD = -.70, p < .02). The dimension of avoidance, which takes on high values in the dismissing attachment style, is expressed in the need for autonomy and a negative vision of others.

### Image of an Ideal Family in Individuals with Different Romantic Attachment Styles

The image of an ideal family, which was evaluated in respondents also through the Semantic Differential Scale, when asked to rate “my family in 10 years”, also found significant differences in respondents with different romantic attachment styles.

#### Comparison of Securely Attached Individuals with Avoidant Ones

Individuals with anxious-avoidant attachment see their ideal family as signiicantly darker (HSD = -.82, p < .01) than those with secure attachment. In addition, they see their ideal family as significantly more unpredictable (HSD = -.75, p < .01). This combination reflects both dimensions of attachment insecurity manifested in this type: anxiety about interpersonal relationships gives a sense of instability and unpredictability of relationships, even in an ideal model, while a negative vision of the future expresses the need for individualization and distance from the family.

#### Comparison of Securely Attached Individuals with Dismissing Ones

Individuals with dismissing-avoidant attachment see their ideal family as signii-cantly darker (HSD = -.85, *p <* .00), colder (HSD = -.59, *p <* .05), and less predictable (HSD = - .67, p < .04) than securely attached persons. Dismissing attachment is characterized by a general adverse perception of others, as evidenced by the two-dimensional model of individual diferences in romantic attachment. In this case, it is expressed in a generally more distant attitude towards family life, which is the comfort zone of dismissing attachment.

#### Comparison of Securely Attached Individuals with Ambivalent Ones

Individuals with ambivalent attachment perceive the ideal family as signiicantly colder (HSD = -.74, p < .05) than securely attached individuals. Th is is due to high anxiety in interpersonal relationships, where there is an extremely pronounced need for emotional closeness. This attachment style is characterized by an increased need for attention and approval from loved ones.

### Image of the Parental Family in Individuals with Different Romantic Attachment Styles

Representations of the parental family, also assessed using the Semantic Differential, did not reveal signiicant diferences depending on the romantic attachment style.

### Similarities and Differences in Family Images

The use of the Semantic Differential allowed us to introduce an additional variable: the sum of the squares of the subjective distances between two objects, which reflects how diferent in our views the two evaluated constructs are, that is, in our case it will be the difference between the images of our own and ideal families, the images of our own and parental families, images of ideal and parental families. We performed a correlation analysis (Spearman’s r) to identify the relationship of these variables with measurements of attachment insecurity: whether there is a correlation between the similarity of these images with anxiety or avoidance in attachment.

The connection between attachment avoidance and the difference between the image of one’s own and the ideal family (r = .17, p < .01) is explained by the desire of such individuals for independence and autonomy, and therefore by their detachment from the family. Attachment anxiety was found to be associated with all three variables, reflecting general anxiety about interpersonal relationships, although individual manifestations are also interesting. The connection between attachment anxiety and the diference between the image of one’s own and the ideal family (r = .22, p < .00) is explained by anxiety in general regarding interpersonal relations, as well as by a tendency to idealization. Attachment anxiety relects a diference between the image of one’s own family and that of the parental family (r = .22, p < .00). This is explained both by anxiety in interpersonal relationships in general, and by the experience of the uniqueness of each relationship, a tendency to idealize one’s own. The tendency toward idealization, characteristic of the attachment anxiety dimension, is enhanced in the image of the ideal family, thereby distinguishing it from all relationships (r = .17, p < .01).

### Orienting Image of Attachment

#### Similarities and Differences in the Images of the Self and Significant Others (Spouse, Mother, and Father)

We tested the assumptions about the importance of the image of a parent of one’s own or the opposite sex for romantic attachment to the signiicant other image using correlation (Spearman’s r), analysis of variance (ANOVA, Tukey’s HSD test) and regression (linear regression) analysis, including using the sum of squares of subjective distances between images of the self and the spouse, images of the self and the mother, images of the self and the father, images of the spouse and the mother, images of the spouse and the father.

In women, the fi gure of the mother and its closeness to the self-image (r *=* .16, *p <* .05) appears to be associated with attachment anxiety in women. The difference between the image of the self and the image of the mother turns out to be subconsciously frustrating and forms anxiety in attachment. Both measures of insecurity of attachment in women (r = .21, *p <* .01 for anxiety; *r* = .19, *p <* .02 for avoidance) are positively correlated with the sum of squares of subjective distances between the mother and the spouse images. The greater are anxiety and avoidance, the greater is the difference between the image of the mother and the spouse. Secure attachment (that is, the absence of high scores for the two dimensions of attachment insecurity) will relect the familiar pattern of love and acceptance in relationships that trusted individuals repeat in a relationship. Thus, women look for patterns in a romantic partner that are similar to their mother-child relationship experience.

In men, the greater is the difference between the image of the spouse and the image of the mother (*r* = .27, *p <* .02), the greater is the attachment avoidance in men. This pattern confirms the importance of the image of the mother for romantic relationships in men, expressed in the common opinion that men prefer women who look like their mothers.

In the whole sample, the sum of the squares of the subjective distances between the images of the self and the spouse is signiicantly higher in individuals with anxious-avoidant attachment than in securely attached individuals (HSD = -24.63, *p <* .01). Anxious-avoidant attachment is characterized by high values on both scales of the two-factor model: these individuals are not ready for intimacy with a partner, and see themselves in a negative way, which also reinforces the difference between the images of self and the spouse.

#### Attachment Continuity Hypothesis

Based on the assumption of the continuity of childhood attachment to a close adult in relation to romantic attachment, we tested a number of hypotheses about the primacy of the image of the parent of one’s own and the opposite sex in relation to the dimensions of the insecurity of romantic attachment, anxiety and avoidance, since the image of a signiicant other as part of an internal working model is introjected in childhood in interaction with a close adult. Through regression model building, we checked whether the image of the opposite-sex parent is indeed a predictor of romantic attachment dimensions, or if the same-sex parent’s image has greater influence. Accordingly, we tested the models where the predictors of anxiety or avoidance were qualities that describe the image of the father or mother, separately for men and women, as well as for the sample as a whole, for a total of 12 models (two dimensions of attachment insecurity (anxiety and avoidance) x two parental images x three categories of the sample (men, women, the whole sample)). Thus, we obtained three models that have statistical signiicance: the image of the mother as a predictor of attachment anxiety in women (R^2^ = .12, F = 2.01, *p <* .04), the image of the father as a predictor of attachment anxiety in women (R2 = .12, F = 2.13 , *p <* .03), the image of the father as a predictor of attachment avoidance in men (R2 = .25, F = 2.02, *p <* .05).

The model in which the anxiety of romantic attachment in women is explained through the image of the mother (R2 = .12, F = 2.01, *p <* .04), has two statistically significant characteristics: measurements of the mother’s image on the light-dark scales (β = -.297, *t* = -2.74, *p <* .01) and “warm - cold” (β = .341, *t* = 2.79, *p <* .01). Thus, a “dark” and “cold” mother is a prerequisite for anxiety in romantic attachment, that is, a negative self-image. This is extremely important because it echoes the process of accepting and developing one’s own femininity, which occurs through identiication with the parent of the same sex. Rejection of the mother in women this way creates self-doubt and an increased need for intimacy and acceptance on the part of a romantic partner.

The model in which the anxiety of romantic attachment in women is explained through the image of the father (R2 = .12, F = 2.13, *p <* .03), has one statistically significant characteristic: the measurement of the father image on the “warm - cold” scale (β = .372, *t* = 3.87, *p <* .00). A “cold” father causes romantic attachment anxiety in women. Th e experience of a warm emotional relationship with the father helps women to successfully communicate with men, as it provides an experience of acceptance and security.

The model in which the avoidance of romantic attachment in men is explained through the image of the father (R2 = .25, F = 2.02, *p <* .05), has two statistically signifi -cant characteristics: measurements of the father image on the “reliable - unpredictable” scale (β = .495, *t* = 2.10, *p <* .04) and “calm - restless” (β = -.33, *t* = -2.07, *p <* .04). It turns out that an unpredictable but calm father’s patterns predict avoidance in men. In this case, we observe a combination of two important manifestations: the instability and changeability of the father’s behavior against the background of external calmness and, possibly, even minor emotional manifestations. Such a context of relations, where the norm is the detachment and variability of the father, frightens and alienates, respectively; an image of the father’s inaccessibility for the family (and most importantly for the mother) is developed, as well as the need for autonomy in interpersonal relationships and the fear of intimacy.

## Discussion

The study of the image of the family in adults with different types of romantic attachment set the main goal of identifying the diferences in this image and showing the inluence of attachment on the image of the family as a system of unconscious attitudes, ideas, and expectations regarding family life in general, which has an individualized personal character by analogy with the image of the world, a concept previously developed in the cultural-historical approach.

In our study, we did not find differences in the image of the parental family depending on the attachment style, contrary to our hypothesis. We may assume that such results may be related to the fact that attachment anxiety is associated with a negative self-image — that is, such individuals project negative patterns of relationships onto themselves and potentially blame themselves for dysfunctional communication. At the same time, attachment avoidance, as a repression of the need for close relationships and lack of interpersonal trust, may also be associated with a conditionally neutral image of the parental family — that is, having no statistically significant differences for different attachment styles. Nevertheless, a systematic review by River et al. (2022) suggests that the quality of attachment in parent-child relationships influences dyadic interactions, thereby reinforcing the problem of the influence of partners’ family histories on dyadic adjustment.

The identification of the orienting image of attachment and its study aims to determine the role of the primary caregiver in the process of choosing and establishing relationships with a romantic partner. In particular, we were interested in whether there is a principle of similarity between the self of a parent of the opposite sex and the self of a romantic partner, which relects psychoanalytic ideas, or whether the image of the primary caregiver, which in Russian culture traditionally refers to the mother, is more important, and also if there are gender diferences in this process. Our study emphasized the image of the mother as an orienting image of attachment for both men and women. The greater difference between the image of the significant other and the image of the mother was associated with both dimensions of attachment insecurity in women, and avoidance in men. Furthermore, the qualities describing both the mother and father images contribute to attachment anxiety in women, and the qualities of the image of the father contributed to attachment-related avoidance in men. Our results are consistent with a longitudinal study by Dugan et al. (2025), which found that the quality of relationships with the mother in childhood is a significant predictor of individual differences in attachment in adulthood. This inding is strengthened by the fact that attachment preferences for parents remain stable over time, thus highlighting the inluence of primary attachment on romantic relationships (Umemura et al., 2018). The quality of relationships with parents inluences the experience of closeness and support in a couple: some research on adolescents has shown that an authoritative relationship quality with their parents is associated with a higher experience of support in a couple (Hadiwijaya et al., 2020). However, relationships with friends also partially mediate the relationship between perceptions of relationships with parents and with a romantic partner (van Rijn-van Gelderen et al., 2025). Nevertheless, we would like to emphasize again that the relationship between attachment patterns in parent-child relationships and romantic attachment in adulthood is ambiguous (Fraley, 2019). Specifically, a number of studies suggest that insecurity patterns diminish with age, and this may manifest diferently for generalised and speciic attachment representations.

## Conclusion

In this study, we combined a cultural-historical approach with attachment theory, which postulates the exceptional signiicance of interpersonal experience for personality development. We substantiated concepts of the family image and the orienting attachment image. We found signiicant diferences in family image based on romantic attachment style, and also found that the orienting attachment image is the mother image for both men and women. However, we failed to ind signiicant differences in the perception of the parental family image based on romantic attachment style.

## Limitations

As limitations of this study, it should be noted that the obtained coefficients are relatively small, so we can rather talk about a trend when interpreting the results. This may be related to the substantive aspects of the Semantic Differential — in other words, to the set of adjectives that were used for the study. In addition, this study measured only the type of romantic attachment, and did not take into account the features of generalized and speciic types of attachment. In addition, this study did not take into account the factor of interaction between the attachment styles of romantic partners; thus, further research into the hypothesis of preference for a similar or complementary partner remains for the future.

Furthermore, it is important to note that although researchers imply that attachment is a relatively stable construct across the lifespan, when we discuss the predictive power of attachment patterns from parent-child relationships to adulthood, attention should also be paid to those factors that may also inluence the continuity of these patterns. This point requires special attention in future research.
